# Natural-Product-Derived Antioxidants and DNA Methylation-Based Epigenetic Aging: A Systematic Review of Human Intervention Studies

**DOI:** 10.3390/antiox15091075

**Published:** 2026-08-28

**Authors:** Fatemeh Taktaz, Salar Hafez-Ghoran

**Affiliations:** Laboratory for Functional Foods and Human Health, Center for Excellence in Post-Harvest Technologies, North Carolina Agricultural and Technical State University, NC Research Campus, 500 Laureate Way, Kannapolis, NC 28081, USA; shafezghoran@ncat.edu

**Keywords:** DNA methylation clock, epigenetic aging, natural product-derived antioxidants, polyphenols, omega-3 fatty acids, biological aging, nutraceuticals

## Abstract

DNA methylation clocks provide a tractable molecular readout for testing whether nutritional and natural-product interventions can modify biological aging. Natural-product-derived antioxidants are biologically plausible candidates because they influence redox signaling, inflammation, mitochondrial function, microbial metabolism and epigenetic regulation, yet their effects on DNA methylation-based aging remain difficult to interpret. Here, we systematically synthesize human intervention studies evaluating antioxidant-rich dietary patterns, botanical and food-derived extracts, marine omega-3 fatty acids, multi-component nutraceuticals and related lifestyle-based interventions with DNA methylation-clock outcomes. The available evidence does not support a uniform epigenetic anti-aging effect. Instead, methylation-age responses were clock-specific, exposure-dependent and often most apparent in metabolically or biologically responsive subgroups. Longer randomized or trial-embedded studies provided the most credible signals, whereas small uncontrolled studies mainly generated hypotheses. Future trials should move beyond claims of epigenetic age reversal and test whether objectively verified natural-product-derived antioxidant exposures produce reproducible, mechanistically linked, and clinically meaningful changes in aging biology.

## 1. Introduction

Aging is not a uniform chronological process. Individuals of the same age can differ widely in metabolic health, immune competence, physical function, disease susceptibility, and mortality risk, creating a need for molecular measures that capture biological aging more directly than calendar time. DNA methylation-based epigenetic clocks have become among the most influential of these measures because they estimate age-related molecular variation from methylation patterns across cytosine-phosphate-guanine (CpG) sites. The first widely used clocks were trained to predict chronological age: the Horvath multi-tissue DNA methylation age estimator was developed for application across diverse human tissues and cell types [1], and Hannum et al. developed a blood-based methylation clock that captured age-associated methylation variation in human populations [2]. Later clocks moved closer to clinically meaningful aging phenotypes. DNA methylation PhenoAge was trained against a clinical biomarker-based phenotypic age and predicted morbidity and mortality beyond chronological age [3], whereas DNA methylation GrimAge incorporated methylation surrogates of plasma proteins and smoking exposure and showed strong association with lifespan and age-related disease risk [4]. DunedinPACE further shifted the field from estimating accumulated biological age to estimating the pace of physiological decline [5].

The expanding use of epigenetic clocks has also exposed a central interpretive challenge: clocks are not interchangeable biological outcomes. In 18,859 participants, Mavrommatis et al. compared 14 epigenetic clocks against 174 incident disease outcomes and all-cause mortality, showing substantial differences in disease association across clocks [6]. Longitudinal evidence further suggests that clock trajectories may carry prognostic information, as faster increases in several clocks were associated with higher mortality risk in the CHIANTI cohort [7]. At the mechanistic level, part of the Horvath clock accuracy may arise from quasi-stochastic DNA methylation changes that accumulate with age [8], whereas biological age estimates can rise during acute physiological stress and partially recover after stress resolution [9]. These findings make DNA methylation clocks attractive endpoints for intervention studies, but they also demand caution: an intervention may affect one clock, one biological domain or one time frame without producing a uniform “anti-aging” signal.

Oxidative stress provides a plausible, but not yet clinically proven, mechanistic bridge between environmental exposures, diet, metabolism and epigenetic aging. Reactive oxygen and nitrogen species participate in redox signaling at physiological levels, but persistent redox imbalance can damage macromolecules, alter mitochondrial function and reshape chromatin regulation. Experimental evidence shows that prolonged low-dose hydrogen peroxide exposure can alter global DNA methylation and histone methylation patterns through inhibition of ten-eleven translocation and Jumonji C-domain demethylase activity [10]. Oxidative stress has also been shown to induce persistent changes in nuclear and mitochondrial DNA methylation in stem-cell models [11] and to promote methylation-dependent repression of stress-protective transcriptional programs in aging and redox-deficient cells [12]. Human data are beginning to support this redox-mitochondrial-epigenetic connection: mitochondrial DNA variant burden was associated with older epigenetic age in young adults [13], and integrated mitochondrial DNA and methylation-aging analysis suggest that mitochondrial variation and epigenetic age acceleration may capture related aspects of aging-associated disease susceptibility [14].

Natural-product-derived antioxidants are therefore biologically plausible candidates for modifying epigenetic aging, but the evidence requires careful separation of mechanistic plausibility from clock-based outcomes. Polyphenols, organosulfur compounds, carotenoids, terpenoids, alkaloids, and marine-derived fatty acids can influence antioxidant defense, inflammatory signaling, mitochondrial function, and stress-response pathways. Some also act through epigenetic mechanisms. Sulforaphane increased nuclear factor erythroid 2-related factor 2 (Nrf2) expression through demethylation of CpG sites in its promoter [15] and reduced DNA methyltransferase activity while altering DNA methyltransferase 1 and DNA methyltransferase 3a expression in breast and prostate cancer cell models [16]. Epigallocatechin gallate inhibited DNA methyltransferase activity and reactivated methylation-silenced genes [17], and subsequent work showed reductions in DNA methyltransferase and histone deacetylase expression in methylation-sensitive colorectal cancer cells [18]. These studies establish mechanistic plausibility, but they do not show that natural-product-derived antioxidants slow DNA methylation-based biological aging in humans.

Human intervention evidence is now emerging, but it is already heterogeneous. For example, the DIRECT PLUS randomized controlled trial linked a polyphenol-rich Green-Mediterranean diet containing green tea, walnuts, and Mankai with attenuation of DNA methylation-assessed biological aging over 18 months [19], and a related analysis suggested epigenome-regulatory potential connected to polyphenol exposure and one-carbon metabolism [20]. Other large trials, however, complicate a simple anti-aging interpretation. In the DO-HEALTH trial, omega-3 supplementation produced small protective effects across selected DNA methylation clocks over three years [21], whereas the COSMOS randomized clinical trial found no significant effect of cocoa extract on five epigenetic aging clocks despite its polyphenol-rich composition [22]. These divergent findings suggest that intervention composition, dose, duration, population characteristics, baseline biological age, and clock selection may determine whether natural-product-derived antioxidants produce measurable epigenetic-aging effects.

This uncertainty defines the gap addressed by the present review. Natural-product-derived antioxidants are widely studied for redox and epigenetic activity, yet most supporting evidence comes from preclinical models, gene-specific methylation studies or conventional oxidative-stress biomarkers. Human clock-based evidence is scattered across dietary-pattern trials, botanical or food-derived interventions, nutrient-like compounds and multi-component regimens. Although a recent systematic review examined nutrition strategies affecting DNA methylation and epigenetic clocks, its scope included broad dietary patterns, methyl-donor nutrients, global DNA methylation and mixed nutritional interventions, without focusing specifically on natural-product-derived antioxidants [23].

The present systematic review asks a more specific question: do human intervention studies provide coherent DNA methylation-clock evidence that natural-product-derived antioxidants can slow or modify epigenetic aging? To address this question, we identify the intervention classes studied, summarize effects on epigenetic age and age acceleration, compare responses across clocks, and evaluate whether clock changes align with oxidative stress, inflammation, mitochondrial function, or related biological markers.

## 2. Methods

### 2.1. Search Strategy and Study Identification

A systematic literature search was conducted across three electronic databases: PubMed/MEDLINE, Scopus, and the Web of Science Core Collection. The search aimed to identify human intervention studies evaluating natural-product-derived antioxidant exposures in relation to DNA methylation-based measures of epigenetic aging. Searches were conducted from database inception to June 2026, with no publication-year restriction. Additional targeted searches were performed to ensure retrieval of key intervention studies, and the reference lists of eligible articles were manually screened.

The search strategy combined free-text keywords and Boolean operators across three concepts: (i) DNA methylation-based biological aging, (ii) intervention or supplementation studies, and (iii) natural-product-derived antioxidant or dietary bioactive exposures. The core search structure was as follows:

(“DNA methylation age” OR “DNA methylation-assessed biological age” OR “biological age attenuation” OR “epigenetic age” OR “epigenetic aging” OR “epigenetic clock” OR “DNA methylation clock” OR “age acceleration” OR “PhenoAge” OR “GrimAge” OR “DunedinPACE” OR “DunedinPoAm” OR “Horvath clock” OR “Hannum clock”)

AND

(intervention OR trial OR randomized OR placebo OR supplementation OR supplement OR diet OR dietary OR nutrition OR nutritional OR “clinical trial” OR crossover)

AND

(polyphenol OR flavanol OR flavonoid, OR catechin OR cocoa OR “green tea” OR resveratrol OR grape OR pomegranate OR ellagitannin OR urolithin OR berry OR berries OR curcumin OR turmeric OR sulforaphane OR glucoraphanin OR broccoli OR carotenoid OR astaxanthin OR omega-3 OR “fish oil” OR “olive oil” OR “olive polyphenol” OR Mediterranean OR “green Mediterranean” OR “Mediterranean diet” OR “DIRECT PLUS” OR “green-MED” OR Mankai OR botanical OR nutraceutical)

**Figure 1:**
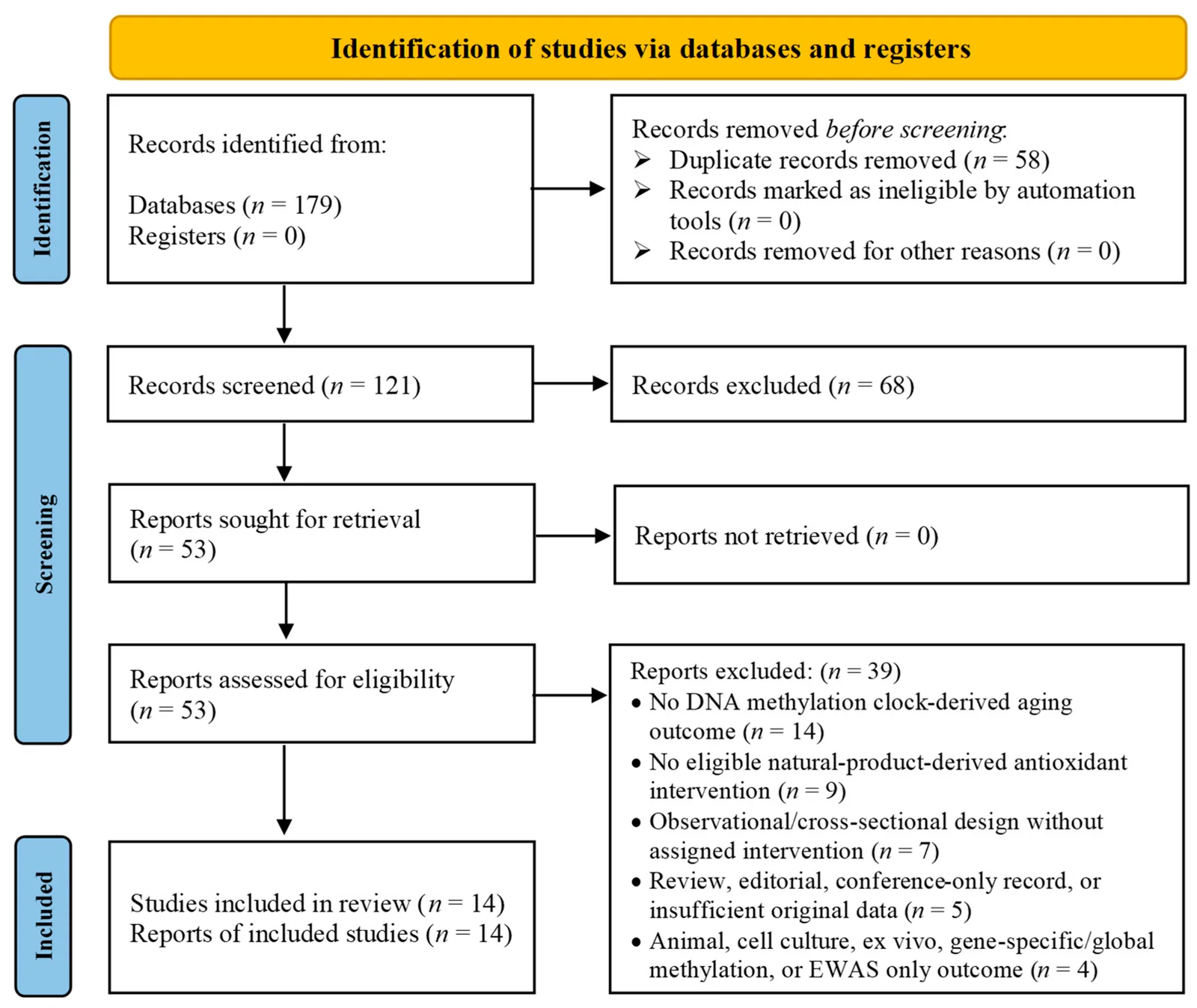
PRISMA 2020 flow diagram of study selection. A total of 179 records were imported from PubMed/MEDLINE, Scopus, and the Web of Science Core Collection. After removal of 58 duplicates, 121 records were screened by title and abstract. Sixty-eight records were excluded, and 53 full-text articles were assessed for eligibility. Thirty-nine full-text articles were excluded, leaving 14 studies for qualitative synthesis. Adapted from the PRISMA 2020 statement.

Search strings were adapted to the syntax of each database. Records were exported and imported into Rayyan for duplicate removal and screening. The study selection and reporting process followed the PRISMA 2020 guidelines [24], including title/abstract screening, full-text eligibility assessment, and reporting of exclusions in a flow diagram. In total, 179 records were imported. After removal of 58 duplicates, 121 unique records were screened by title and abstract. Sixty-eight records were excluded at this stage. Fifty-three full-text articles were assessed for eligibility, of which 39 were excluded. Fourteen studies met the inclusion criteria and were included in the qualitative synthesis. The study selection process is presented in the PRISMA 2020 flow diagram (Figure 1). This review was retrospectively registered with OSF Registries (https://osf.io/vjxhg/overview, accessed on 21 August 2026).

### 2.2. Eligibility Criteria

Studies were considered eligible if they met all of the following criteria: (i) included human participants; (ii) evaluated an assigned dietary, nutritional, botanical, nutraceutical, marine-derived or natural-product-derived antioxidant intervention; (iii) used an intervention design, including randomized controlled trials, crossover trials, non-randomized intervention studies, secondary analyses of intervention datasets or prospective single-arm intervention studies; and (iv) reported at least one DNA methylation clock-derived measure of epigenetic aging.

Eligible interventions included antioxidant-rich foods, polyphenol-rich dietary patterns, botanical extracts, food-derived bioactives, phytochemicals, marine-derived bioactives and nutraceutical formulations. Interventions of interest included, but were not limited to, cocoa flavanols, green tea catechins, resveratrol, berry- or grape-derived products, pomegranate- or ellagitannin-related compounds, urolithin-related interventions, sulforaphane- or glucoraphanin-containing interventions, carotenoids, astaxanthin, olive polyphenols, omega-3 fatty acids, Mediterranean or Green-Mediterranean dietary interventions, and multi-component natural-product-derived supplement combinations.

Eligible outcomes included DNA methylation age, epigenetic age acceleration, intrinsic or extrinsic epigenetic age acceleration, Horvath age, Hannum age, DNA methylation PhenoAge, GrimAge, GrimAge2, DunedinPACE, DunedinPoAm and other DNA methylation-derived biological aging measures. Studies using targeted methylation-age estimators were retained when the outcome was explicitly reported as DNA methylation age or epigenetic aging, but these studies were interpreted separately from genome-wide clock studies.

The following exclusion criteria were applied: (i) animal, cell-culture or ex-vivo studies, (ii) observational or cross-sectional studies without an assigned intervention, (iii) Mendelian randomization studies, (iv) narrative reviews, systematic reviews, editorials, case reports and conference-only records, (v) studies reporting only gene-specific methylation, global methylation, epigenome-wide association studies (EWAS) or other methylation outcomes without a DNA methylation clock-derived aging measure, (vi) studies evaluating general lifestyle or dietary exposure without a clearly assigned intervention.

When duplicate datasets or overlapping analyses were identified, the article providing the most relevant intervention and DNA methylation clock information was retained for synthesis.

### 2.3. Study Selection and Data Extraction

All records were screened according to predefined eligibility criteria. The study selection process followed the PRISMA 2020 guidelines and is illustrated in Figure 1 (PRISMA flow diagram). Titles and abstracts were first reviewed to exclude clearly irrelevant records. Data extraction was performed using a standardized form that captured first author and year of publication, journal and DOI, study design, participant characteristics and health status, sample size included in DNA methylation analysis, intervention type, dose and duration, comparator or control condition, biological sample used for methylation analysis, DNA methylation platform or assay, DNA methylation clock or epigenetic aging measure, main clock-related findings, and secondary inflammatory, oxidative stress, mitochondrial, metabolic, immune, microbiome, body-composition, and physical-function outcomes.

Studies were additionally categorized by intervention class to facilitate structured synthesis. Categories included Mediterranean or polyphenol-rich dietary patterns, botanical or food-derived extracts, marine-derived omega-3 interventions, multi-component natural-product-derived nutraceutical interventions, and indirect or borderline dietary/lifestyle interventions.

### 2.4. Risk-of-Bias Assessment

The methodological quality and interpretability of the included studies were evaluated qualitatively because the evidence base included heterogeneous designs, including randomized controlled trials, randomized trial substudies, secondary analyses of intervention datasets, uncontrolled before-after studies, and single-arm open-label interventions. Because the included studies were heterogeneous in design, intervention type, comparator structure and methylation-clock outcomes, a single formal risk-of-bias instrument was not applied uniformly. Instead, studies were evaluated using a qualitative domain-based framework tailored to intervention-clock interpretability. Each study was assessed according to the following domains:

- Randomization and allocation methods.
- Presence and type of comparator or placebo.
- Blinding.
- Sample size and completeness of DNA methylation data.
- Whether DNA methylation aging was a primary, prespecified secondary, ancillary, post hoc, or exploratory outcome.
- Use of validated genome-wide DNA methylation clocks versus targeted methylation-age estimators.
- Intervention specificity and potential lifestyle co-interventions.
- Commercial involvement or other potential conflicts of interest.
- Consistency of findings across clocks and participant subgroups.

**Figure 2:**
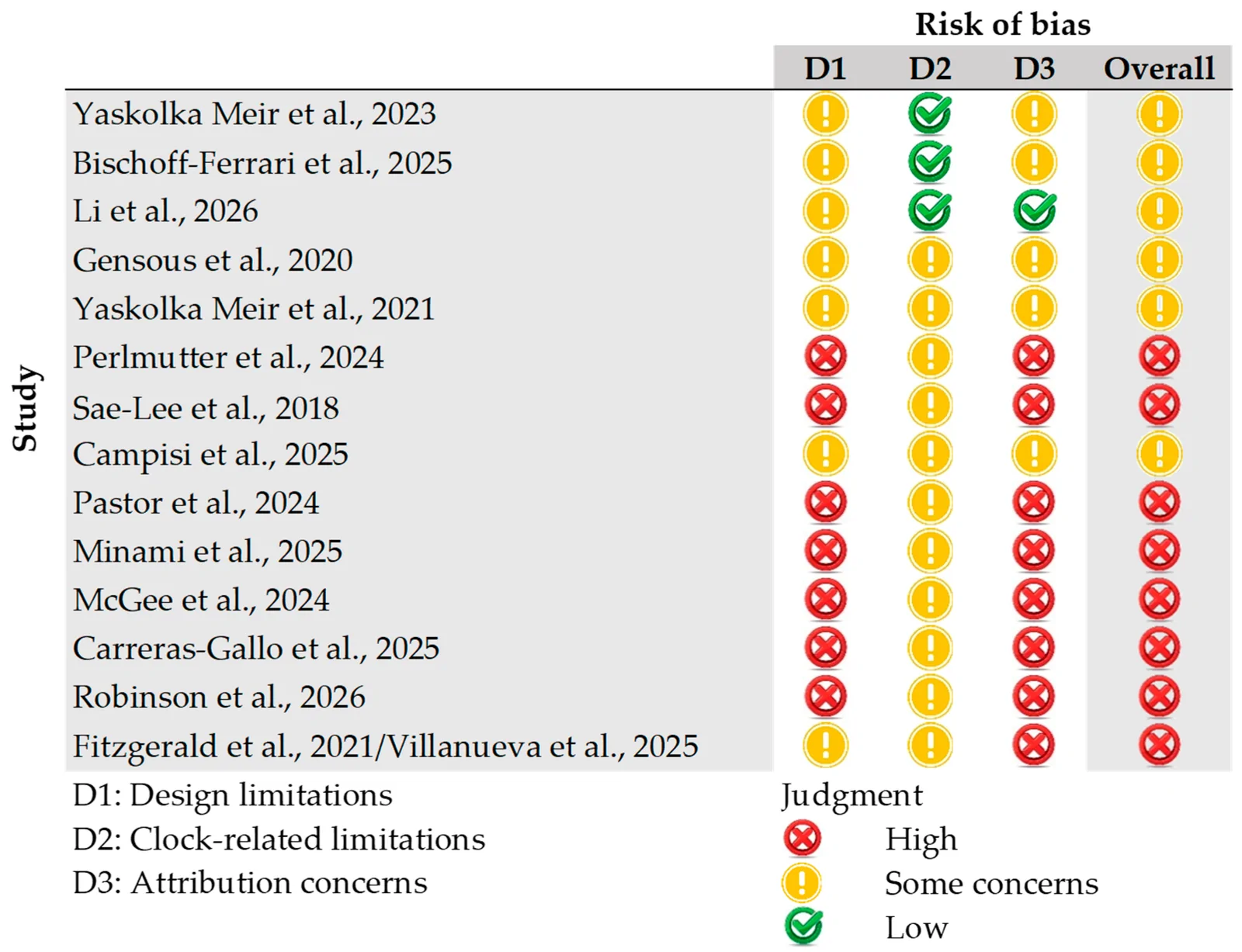
Risk-of-bias considerations for the included human studies based on qualitative assessment [19,21,22,25,26,27,28,29,30,31,32,33,34,35,36].

Overall risk of bias was categorized as low, some concerns or high. The strength of evidence was summarized as strong, moderate, low or very low based on study design, comparator quality, sample size, directness of the intervention-clock relationship, consistency across DNA methylation clocks, and whether findings reflected whole-cohort effects or subgroup-specific associations. A summary of the qualitative risk-of-bias assessment across the included studies is shown in Figure 2, whereas detailed study-level judgments and qualitative certainty ratings are provided in Table S1.

### 2.5. Data Synthesis

Given the heterogeneity of study designs, populations, intervention types, durations, biological samples, methylation platforms, DNA methylation clocks, and outcome reporting, a narrative synthesis was performed. The extracted data were summarized qualitatively and organized into intervention domains:

- Mediterranean and polyphenol-rich dietary patterns.
- Botanical and food-derived extracts.
- Marine-derived omega-3 interventions.
- Multi-component natural-product-derived nutraceutical interventions.
- Indirect or borderline dietary/lifestyle interventions.

Clock outcomes were interpreted according to their biological training target. Chronological-age-trained clocks were distinguished from morbidity- and mortality-associated clocks and from pace-of-aging measures. Greater interpretive weight was given to findings from randomized designs, comparator-supported effects, consistency across biologically relevant clocks, and concordance with inflammatory, metabolic, oxidative-stress, mitochondrial, immune, microbiome, or body-composition outcomes.

The PRISMA 2020 Checklist is provided in the Supplementary Materials (Table S2) to ensure methodological transparency.

## 3. Evidence Synthesis of Human Intervention Studies

The 14 included studies represented heterogeneous human intervention evidence on whether natural-product-derived antioxidant exposures can modify DNA methylation-based measures of biological aging. Interventions ranged from polyphenol-rich dietary patterns and botanical or food-derived extracts to marine omega-3 fatty acids and multi-component nutraceutical formulations. The studies also differed in age, health status, metabolic risk, comparator design, biological sample, methylation platform and clock selection, limiting direct comparison across interventions. In most studies, DNA methylation age was assessed as a secondary, ancillary or exploratory outcome rather than as the primary clinical endpoint. Table 1 summarizes the study design, population, intervention characteristics, methylation methods, clock outcomes and main findings.

### 3.1. Polyphenol-Rich Dietary Patterns and Whole-Diet Interactions

Mediterranean and polyphenol-rich dietary pattern interventions provide the most integrated human evidence for diet-related modulation of DNA methylation-based aging, but they also complicate attribution because polyphenol exposure is embedded within a broader nutritional matrix that affects adiposity, liver fat, inflammation, oxidative stress, microbiome-derived metabolites and one-carbon metabolism. This complexity is consistent with recent clinical-trial syntheses showing that nutrition effects on DNA methylation and epigenetic clocks are heterogeneous and strongly dependent on intervention design, population characteristics and clock selection [23]. Mechanistically, Mediterranean-type diets are relevant to epigenetic aging because they supply polyphenols, unsaturated fatty acids, fiber and micronutrients that can influence oxidative-stress signaling, inflammatory pathways and epigenetic regulation [37]; clinically, this dietary pattern has established cardiometabolic relevance in randomized-trial settings [38], and higher Mediterranean-diet polyphenol intake has been associated with lower inflammatory biomarkers in the PREDIMED trial [39]. Within the included clock-based intervention evidence, the NU-AGE epigenetic substudy showed that a 1-year Mediterranean-like diet in 120 European older adults aged 65–79 years from the NU-AGE project including 60 participants recruited in Italy and 60 recruited in Poland, produced a trend toward Horvath-clock-based epigenetic rejuvenation, with significant reductions in epigenetic age acceleration and intrinsic epigenetic age acceleration mainly in Polish women, whereas extrinsic epigenetic age acceleration was unchanged [25]. This suggests a possible cell-intrinsic methylation-aging response, but the country- and sex-specific signal indicates that population background, baseline epigenetic age and host factors may modify the intervention effect. Because the strongest signal was observed in a country- and sex-specific subgroup, these findings should not be interpreted as a generalized Mediterranean-diet effect.

The CENTRAL MRI substudy provided a complementary metabolic interpretation: in 120 adults with abdominal obesity or dyslipidemia, predominantly men (110 men and 10 women), recruited from a research-center workplace in Dimona, Israel, 18 months of low-fat or Mediterranean/low-carbohydrate lifestyle intervention did not produce a significant diet-arm difference in methylation-age change, but methylation aging was attenuated among participants who achieved > 5% weight loss and healthy intrahepatic fat status [26]. Thus, in metabolically at-risk populations, methylation-age attenuation may reflect downstream cardiometabolic remodeling rather than assigned dietary pattern alone. The strongest polyphenol-linked signal came from DIRECT PLUS, in which 256 participants with abdominal obesity or dyslipidemia, recruited from an isolated workplace at the Nuclear Research Center Negev in Dimona, Israel, and comprising 228 men and 28 women, were assigned to healthy dietary guidelines, Mediterranean diet, or a polyphenol-rich Green-Mediterranean diet enriched with walnuts, green tea, and Mankai for 18 months. Although no broad between-group effect was observed across the full clock panel, greater Green-Mediterranean adherence was associated with attenuation of Li and Hannum methylation age, and these changes tracked with urinary polyphenol-related metabolites, including hydroxytyrosol, tyrosol and urolithins [19]. The small number of women in DIRECT PLUS should also be considered when interpreting sex-specific generalizability. A related DIRECT PLUS multi-omics analysis further supports biological plausibility, linking the Green-Mediterranean diet to epigenetic regulatory potential and one-carbon metabolism [20]. These studies support a context-dependent model in which methylation-age attenuation is most interpretable when sustained polyphenol exposure is supported by adherence biomarkers and accompanied by improvements in adiposity, liver fat or metabolic risk.

### 3.2. Botanical and Food-Derived Extracts

Compared with whole-diet interventions, studies of botanical or food-derived extracts offer more compositionally defined antioxidant exposures but have produced less consistent clock-based evidence, largely because most were small, exploratory, uncontrolled or used targeted methylation-age estimators rather than genome-wide clocks. Mechanistically, this class is plausible because plant polyphenols can influence redox-sensitive signaling, DNA methyltransferase activity, histone modifications, microRNA expression, inflammatory pathways and gut microbiome-derived metabolites [27,40]. However, the strongest controlled evidence did not support a clear epigenetic aging effect for an isolated flavanol intervention. In the prespecified COSMOS ancillary study, 2 years of cocoa extract providing 500 mg cocoa flavanols per day, including 80 mg (−)-epicatechin, had no significant effect on PCHannum, PCHorvath, PCPhenoAge, PCGrimAge or DunedinPACE among 958 older adults, whereas the multivitamin-multimineral arm modestly slowed PCGrimAge and PCPhenoAge [22]. This large null finding is important because cocoa flavanols have cardiometabolic and vascular biological plausibility, yet these effects did not translate into measurable clock attenuation in a generally healthy older trial subset. Earlier secondary analysis of a small monomeric and oligomeric flavanol dataset similarly found no significant whole-cohort change in Horvath DNA methylation age, although the same study showed that folic acid plus vitamin B12 reduced epigenetic age acceleration only in women with the methylenetetrahydrofolate reductase 677CC genotype, underscoring genotype- and pathway-specific nutritional effects [28]. More positive signals came from smaller botanical or food-matrix studies. In a randomized, double-blind, placebo-controlled trial, 12 weeks of *Monarda didyma* L. extract stabilized a targeted five-marker DNAmAge estimator based on ELOVL2, C1orf132, krüppel-like factor 14, tripartite motif-containing protein 59 and four and a half LIM domains protein 2, while the placebo group showed accelerated epigenetic aging and hypermethylation of ELOVL2 and FHL2; this was supported by preclinical evidence of reduced protein oxidation, telomere shortening and DNA damage, but remains less directly comparable with genome-wide clock studies [29]. In an open-label pilot study, a Tartary buckwheat-derived polyphenol supplement for 90 days altered selected epigenetic-age and immune-cell markers, including subgroup-specific changes in PCPhenoAge, PCGrimAge and OMICmAge, but the absence of placebo control and heterogeneity of individual clock responses limit causal interpretation [27]. Similarly, a prospective before-after study reported reduced DNAm PhenoAge and more negative epigenetic age acceleration after approximately 3.5 months of resveratrol-enriched Malbec wine, together with improved fat and muscle mass, but the small sample size, lack of control group and alcoholic matrix make this evidence hypothesis-generating rather than confirmatory [30].

### 3.3. Marine-Derived Omega-3 Interventions

Marine-derived omega-3 fatty acids represent a mechanistically distinct class of natural-product-derived bioactives because their putative effects on epigenetic aging are more likely mediated through anti-inflammatory lipid signaling, membrane remodeling, specialized pro-resolving mediators and immunometabolic regulation than through direct radical-scavenging activity. Eicosapentaenoic acid (EPA) and docosahexaenoic acid (DHA) can alter cell-membrane fatty-acid composition and generate resolvins, protectins and maresins, providing a plausible link between marine lipid exposure, inflammation resolution and aging biology [41,42]. Omega-3 fatty acids have also been associated with DNA methylation changes in immune and inflammatory pathways, supporting their relevance to epigenetic regulation [43,44]. Among the included studies, the strongest evidence came from the DO-HEALTH Bio-Age analysis, a post hoc methylation substudy of a randomized factorial trial in 777 generally healthy Swiss adults aged ≥ 70 years. Over 3 years, participants received vitamin D at 2000 IU per day, marine omega-3 fatty acids at 1 g per day, a simple home exercise program, or combinations of these interventions. Omega-3 supplementation alone slowed PhenoAge, GrimAge2 and DunedinPACE, while combined omega-3, vitamin D and exercise showed an additive benefit for PhenoAge; the standardized effects corresponded to approximately 2.9–3.8 months of biological-age attenuation over 3 years [21]. A second lower-certainty study examined public longitudinal methylation data from 25 patients with mild cognitive impairment and 20 controls who received vitamin D and/or marine omega-3 fatty acid supplementation for 2 years. In patients with mild cognitive impairment, PhenoAge and GrimAge decelerated after the intervention, with accompanying changes in natural killer cell estimates [31]. However, the small sample size, secondary use of public data, disease-specific population, and inability to isolate omega-3 from vitamin D limit causal interpretation. Overall, marine omega-3 supplementation provides one of the more credible signals in the current evidence base, particularly for morbidity-, mortality- and pace-of-aging-related clocks, but it remains unclear whether these effects are separate from vitamin D, exercise, immune-cell shifts or baseline inflammatory and metabolic status.

### 3.4. Multi-Component Natural-Product-Derived Nutraceutical Interventions

Multi-component nutraceutical interventions are designed to target several aging pathways simultaneously, including oxidative stress, inflammation, mitochondrial dysfunction, nutrient sensing, telomere maintenance and epigenetic regulation, a rationale consistent with the updated hallmarks-of-aging framework [45]. However, this same multi-target design limits causal interpretation, because clock changes cannot be attributed to a single antioxidant, nutrient or biological pathway, and longitudinal clock analyses are particularly vulnerable to small sample size, assay variability and clock-specific responsiveness [23,46]. In an uncontrolled open-label study of 80 healthy adults aged ≥ 60 years, 12 weeks of a combined supplement containing vitamin B3, vitamin C, vitamin D, omega-3 fish oil, resveratrol, olive fruit phenols and astaxanthin did not change whole-cohort blood-based Horvath, Hannum, DNAm PhenoAge, GrimAge or mean epigenetic age, nor saliva-based InflammAge. A signal emerged only in participants with baseline epigenetic age acceleration ≥ 2 years, who showed reduced saliva InflammAge and InflammAge acceleration, while C-reactive protein (CRP) decreased only in the subgroup with elevated baseline high-sensitivity CRP; growth differentiation factor 15 (GDF-15) was unchanged [32]. These findings suggest possible benefits in biologically older or more inflamed individuals, but the lack of placebo control and subgroup-restricted effects make the evidence low certainty. A broader hallmarks-of-aging-targeted formulation was tested in a 12-month single-arm trial of 51 adults receiving the SRW Cel System nutraceutical range alongside recommendations for daily walking and mindfulness. The study reported improvements in grip strength, chair-stand performance and body composition, but no clear effect on systemic inflammation measured by CRP or interleukin-6. Epigenetic outcomes were mixed: PC-Horvath pan-tissue age and DNAmGrip decreased, DamAge decreased transiently at 3 and 6 months, but PC-Horvath skin/blood and DunedinPACE increased by 12 months, while several other clocks or biomarker proxies were unchanged [33]. Thus, despite favorable functional signals, the discordant clock responses, single-arm design, commercial formulation and lifestyle co-intervention prevent firm conclusions about epigenetic age reduction. Finally, in a short randomized crossover trial of adults aged 50–65 years with obesity, 30 days of a greens-based supplement increased Horvath epigenetic age, whereas PCGrimAge, AdaptAge and DamAge showed no significant intervention effect; gut microbial alpha diversity, metabolic biomarkers, body composition, actigraphy and psychological outcomes were also largely unchanged [34]. These findings are best interpreted as hypothesis-generating: selected subgroups or selected clocks may respond, but current studies do not show reproducible whole-cohort slowing of DNA methylation-based aging, and future trials require placebo-controlled, blinded designs, biomarker-confirmed adherence, adequate methylation sample size and prespecified clock endpoints.

### 3.5. Indirect and Borderline Dietary/Lifestyle Interventions

One included study was classified as an indirect or borderline dietary/lifestyle intervention because the antioxidant-supportive diet and lifestyle program was tested as part of a broader multimodal intervention, not as a discrete natural-product-derived antioxidant. The parent Methylation Diet and Lifestyle pilot randomized clinical trials assigned healthy men aged 50–72 years to an 8-week multimodal intervention that combined a plant-centered diet, exercise, sleep guidance and meditation, and reported a 3.23-year between-group reduction in Horvath 2013 epigenetic age compared with usual care [35]. The secondary analysis by Villanueva et al. specifically examined which dietary components might explain variability in epigenetic-age response and found that foods categorized as “methyl adaptogens”-green tea, oolong tea, turmeric, rosemary, garlic, and berries-were significantly associated with reduced epigenetic age after adjustment for weight change and baseline epigenetic age acceleration [36]. These foods provide polyphenols and related phytochemicals capable of modulating DNA methyltransferase, redox-sensitive signaling and inflammatory pathways, but the study cannot isolate antioxidant effects from the broader intervention context. Weight loss did not significantly predict epigenetic-age reduction in the adjusted model, suggesting that the dietary signal was not solely explained by short-term weight change; the sample was small and demographically homogeneous; dietary intake was self-reported; and epigenetic aging was assessed only with the Horvath pan-tissue clock in saliva [36]. Nevertheless, the evidence remains of low certainty because the analysis was exploratory, the sample was small and demographically homogeneous, dietary intake was self-reported, and epigenetic aging was assessed only with the Horvath pan-tissue clock in saliva.

Across intervention classes, favorable clock responses were inconsistent. The most credible signals came from longer randomized or trial-embedded studies, whereas uncontrolled botanical and multi-component nutraceutical studies were mainly hypothesis-generating. Detailed study-level risk-of-bias judgments and qualitative certainty ratings are provided in Table S1.

## 4. From Clock Change to Biological Meaning: Causal, Mechanistic, and Responder Gaps

**Figure 3:**
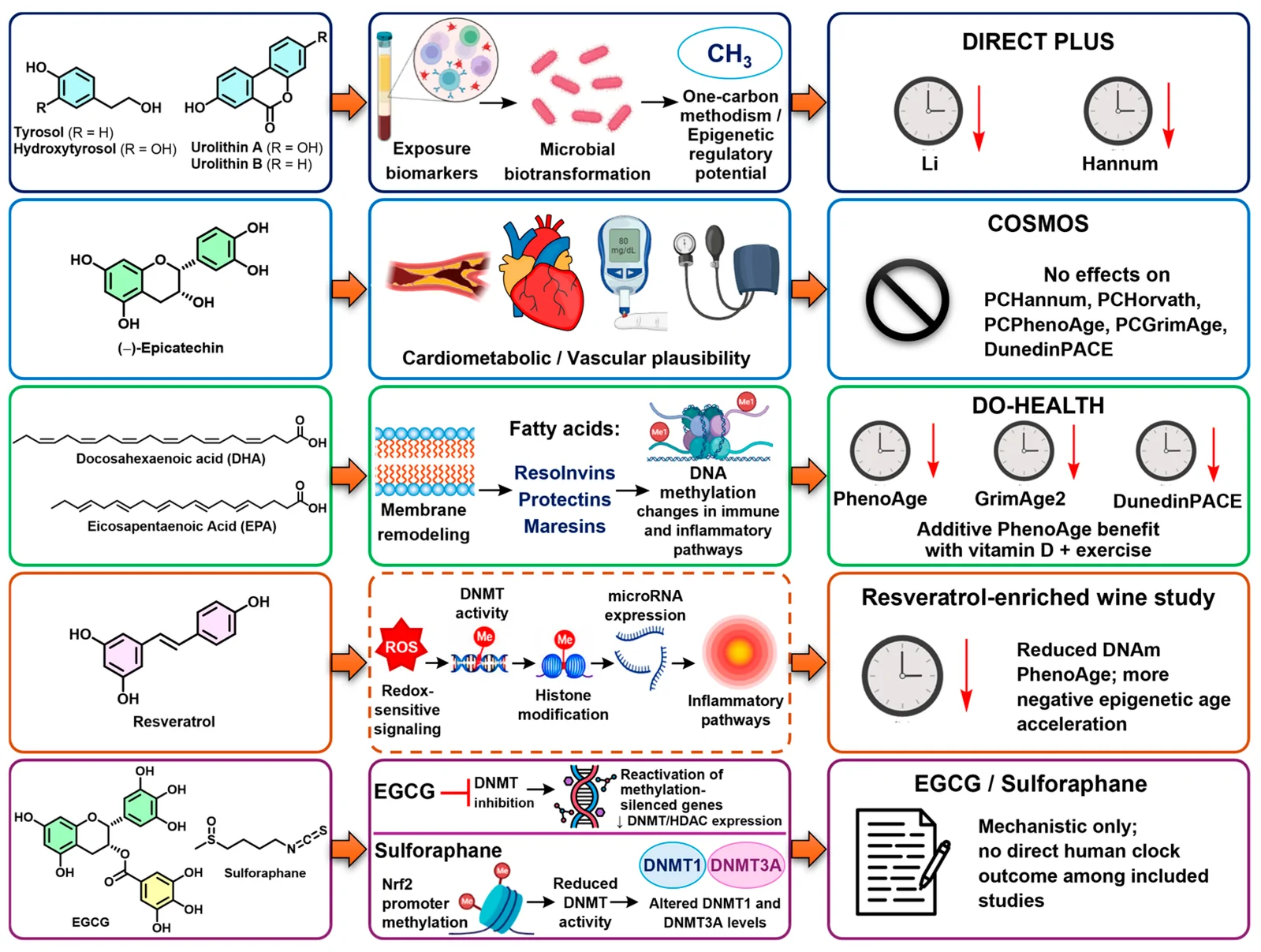
Representative bioactives, mechanistic pathways and selected DNA methylation-clock outcomes. The figure links selected natural-product-derived antioxidant exposures with plausible biological pathways and corresponding clock-related findings. Dashed borders indicate broader mechanistic context rather than direct compound-specific mechanisms demonstrated in the included human intervention studies. Red arrows indicate reduction in the reported DNA methylation clock.

A major gap in this field is the distance between observing a DNA methylation-clock change and demonstrating that an intervention has modified aging biology in a meaningful way. Aging biomarkers are increasingly used to screen candidate longevity interventions, but their interpretation depends on context of use, analytical reliability, biological responsiveness, and evidence that biomarker changes are linked to health-relevant outcomes [47,48]. This distinction is particularly important for natural-product-derived antioxidant trials because small changes in epigenetic age may reflect short-term shifts in inflammation, immune-cell composition, nutrient status or tissue sampling rather than durable slowing of organismal aging. Recent geroscience endpoint work similarly emphasizes that trials targeting aging biology should connect biomarker movement to functional, clinical or disease-risk outcomes, rather than relying on molecular endpoints alone [49]. Figure 3 illustrates how representative bioactives, mechanistic pathways and selected DNA methylation-clock findings are connected across the included intervention evidence.

### 4.1. Causal Specificity

The first interpretive gap is causal specificity. Most natural-product interventions in this review cannot be reduced to “antioxidant activity”, because the tested exposures often combine polyphenols, micronutrients, fatty acids, fiber, methyl donors, microbial substrates and broader dietary change. This matters because nutritional biomarkers are still unevenly validated, and self-reported intake alone is not sufficient to confirm exposure to the bioactive compounds presumed to drive methylation changes [50]. Diet-related metabolomics can strengthen causal interpretation by linking intervention assignments to measurable exposure signatures in blood or urine, but methodological heterogeneity remains a challenge across feeding and intervention studies [51].

### 4.2. Mechanistic Resolution

The second interpretive gap is mechanistic resolution. Polyphenols and other natural-product-derived compounds may influence methylation-aging measures through antioxidant effects, but they may also act through anti-inflammatory signaling, endothelial function, mitochondrial stress responses, gut microbial biotransformation, and one-carbon metabolism. A recent systematic review of 153 human studies showed substantial inter-individual variability in the absorption, distribution, metabolism and excretion of phenolic metabolites, indicating that nominally similar polyphenol exposure can produce very different internal metabolite profiles across participants [52].

### 4.3. Responder Biology

The third gap is responder biology. Current studies are rarely powered to identify who benefits, yet this may be the most important question for the field. Polyphenol trials increasingly recognize substantial interindividual variability in clinical and metabolic responses, driven by baseline diet, genetics, microbiome composition, metabolic health, sex, age, and medication use [52,53]. In epigenetic-aging studies, responder profiles may include individuals with accelerated baseline epigenetic age, higher inflammatory burden, poorer metabolic health, low baseline nutrient status or specific microbiome-derived metabolite capacities. Without prespecified responder analysis and objective exposure biomarkers, subgroup findings remain difficult to distinguish from regression to the mean, chance findings, or selective interpretation.

### 4.4. Clock Selection and Endpoint Interpretation

A related endpoint-level issue is that the field lacks consensus on which methylation clocks are appropriate endpoints for nutrition and natural-product interventions. Epigenetic clocks differ in training targets, tissue performance and clinical meaning, and recent reviews emphasize that many clocks are not directly interchangeable across aging, mortality and disease-risk applications [54]. Cross-tissue work further shows that clock estimates can vary by sample type, which is especially relevant when trials use blood, saliva or buccal samples interchangeably [55]. Recent critiques also caution against moving epigenetic clocks too quickly into individual-level clinical interpretation without stronger evidence of clinical utility [56]. Thus, future antioxidant trials should not simply test many clocks and highlight the favorable one; they should prespecify clock endpoints based on biological rationale, align them with mechanistic outcomes, and report discordant clock responses transparently.

## 5. Discussion and Perspective

This review reframes natural-product-derived antioxidants as candidate modulators of nutrition-sensitive aging biology rather than as established epigenetic rejuvenation interventions. The evidence synthesized here shows that DNA methylation clocks can register responses to some dietary, botanical, marine-derived, and nutraceutical exposures, but these responses are too heterogeneous to support a general anti-aging claim. A more useful interpretation is that natural-product-derived-antioxidant interventions may help identify which biological systems remain modifiable in midlife and older age. Human multi-omics studies show that aging does not progress as a single uniform trajectory: plasma proteomic profiles change in nonlinear waves across the lifespan, and longitudinal deep phenotyping has identified individualized “ageotypes” involving metabolic, immune, hepatic and renal pathways [57,58]. This system-level view fits the findings of the present review better than a single-clock, single-antioxidant model.

The next conceptual step is to move from product-based claims to biology-based trial questions. Instead of asking whether a supplement “reduces biological age”, future studies should ask whether a defined exposure modifies a prespecified aging-relevant system, such as inflammatory tone, postprandial metabolic control, hepatic lipid handling, immune activation, microbial metabolite production or physical resilience. This shift would bring antioxidant epigenetic-aging trials closer to geroscience trial design, where the aim is not merely to move a biomarker but to test whether an intervention modifies common biology linked to multiple chronic diseases and functional decline [59,60]. In this framework, methylation clocks are not the stand-alone endpoint of the trials; they are one layer of evidence within a broader causal chain.

Precision nutrition provides an important model for this transition. Large human studies show that metabolic responses to the same foods vary markedly between individuals, and that person-specific factors, including gut microbiome composition, can improve prediction of postprandial glycemic and lipemic responses [61]. Deep phenotyping studies further show that habitual diet, microbiome features and cardiometabolic biomarkers are tightly interconnected, supporting the view that dietary bioactives should be studied as part of host-microbe-metabolic networks rather than as isolated compounds [62]. This has direct implications for antioxidant trials: two participants assigned to the same polyphenol-rich or marine-derived intervention may have different internal exposures, downstream metabolites and methylation responses.

This perspective also explains why future studies should prioritize mechanistic triangulation over larger exploratory clock panels. Dietary interventions can produce measurable changes in immune and microbiome-related biology, as shown in controlled feeding work where microbiota-targeted diets altered immune status in humans [63]. Similarly, the response to plant-food bioactives is shaped by interindividual variability in absorption, metabolism, gut microbiota, genotype, sex, age and baseline health status [64]. These findings suggest that the most informative antioxidant epigenetic-aging trials will be those that measure the pathway between intake and response: exposure biomarkers, microbial metabolites, inflammatory mediators, metabolic outcomes, immune-cell profiles and functional endpoints alongside DNA methylation clocks.

### Strengths and Limitations

A strength of this review is its focused scope. The analysis moves beyond the broad question of whether nutrition influences DNA methylation and specifically examines whether human intervention studies support a coherent link between natural-product-derived antioxidant exposures and DNA methylation-based aging. Restricting eligibility to human intervention studies strengthened causal relevance compared with observational evidence, although it also narrowed the available evidence base. The synthesis also separated dietary patterns, botanical and food-derived extracts, marine omega-3 interventions, multi-component nutraceuticals and indirect lifestyle-based interventions, allowing the evidence to be interpreted by exposure type, clock family and inferential strength.

The main limitation is the heterogeneity of the underlying evidence. Interventions differed in composition, dose, duration, comparator structure, participant phenotype, methylation tissue, assay platform and clock algorithm. Consequently, a meta-analysis was not appropriate, and the review could not estimate a pooled effect size. Several studies assessed methylation age as an exploratory, ancillary or secondary outcome, and some relied on uncontrolled designs, subgroup analysis or targeted methylation-age estimators. These features limit causal inference and reduce direct comparability across studies.

Although this review was not prospectively registered, retrospective registration was completed with OSF Registries (https://osf.io/vjxhg/overview, accessed on 21 August 2026). Unpublished studies, trial registries and gray literature were not searched systematically. Publication bias and selective outcome reporting therefore cannot be excluded. These limitations mean that the conclusions should be interpreted as a critical evidence map, not a definitive efficacy estimate. The review supports biological plausibility and identifies priorities for future trial design, but it does not establish natural-product-derived antioxidants as reproducible epigenetic anti-aging interventions.

## 6. Conclusions

Human intervention evidence does not yet support natural-product-derived antioxidants as established epigenetic anti-aging interventions. Instead, the available studies indicate a more conditional pattern: selected dietary, botanical, marine-derived and nutraceutical exposures may influence specific DNA methylation-aging measures in biologically responsive contexts. These effects appear to depend on intervention composition, achieved exposure, participant phenotype, tissue sampled and clock selection. The field now needs adequately powered, biomarker-anchored randomized trials that connect methylation-clock changes with mechanistic and functional outcomes. Until such evidence is available, clock-based findings should be interpreted as early signals of nutrition-responsive aging biology, not as definitive evidence of biological-age reversal.

## Figures and Tables

**Table 1:** Characteristics and main findings include human intervention studies evaluating natural-product-derived antioxidant exposures and DNA methylation-based aging outcomes.

Study	Design and Population	Intervention, Duration and Comparator	Sample and Methylation Method	DNA Methylation Aging Outcome(s)	Main Methylation-Clock Finding, Indicating Reported Effect Size/Precision
Yaskolka Meir et al., 2023 [19]	– Randomized dietary intervention; – Adults with abdominal obesity or dyslipidemia (n = 256; 228 men/28 women); recruited from an isolated workplace at the Nuclear Research Center Negev, Dimona, Israel	– Healthy dietary guidelines, Mediterranean diet, or Green-Mediterranean diet for 18 months; – Diet arm comparison	Blood; Illumina EPIC/850K BeadChip methylation array	Li mAge; Horvath 2013; Hannum 2013; Horvath skin/blood; PhenoAge; PCGrimAge, IEAA and DunedinPACE	– No significant between-group intervention effect across clocks. Li mAge change: 1.06 ± 1.98 years in HDG, 1.05 ± 1.97 years in MED and 0.77 ± 2.98 years in Green-MED – Higher Green-MED diet score was associated with lower relative Li mAge change (β = −0.338, *p* = 0.0178; adjusted β = −0.41, *p* = 0.004). MED-style diets showed ~8.9-month lower observed vs. expected Li mAge (64.95 ± 8.67 vs. 65.69 ± 7.91 years, *p* = 0.02), Hannum mAge adjusted β = −0.38, *p* = 0.03
Bischoff-Ferrari et al., 2025 [21]	– Post hoc methylation analysis within a randomized factorial trial; – Adults aged ≥ 70 years (n = 777; mean age ~75 years; 59–60% women)	– Vitamin D 2000 IU/day, marine omega-3 fatty acids 1 g/day and/or simple home exercise program for 3 years; – Factorial control comparison	Whole blood; Illumina Infinium MethylationEPIC; iScan array	Primary clocks: PhenoAge, GrimAge, GrimAge2 and DunedinPACE Secondary clocks: Horvath and Hannum	– Omega-3 slowed PhenoAge, GrimAge2, and DunedinPACE, combined omega-3 + vitamin D + exercise showed an additive benefit for PhenoAge d = −0.16 (95% CI −0.30 to −0.02); GrimAge2 d = −0.32 (−0.59 to −0.06); and DunedinPACE d = −0.17 (−0.31 to −0.04). Combined interventions showed additive benefits for PhenoAge (d = −0.24 to d = −0.32)
Li et al., 2026 [22]	– Prespecified ancillary analysis of the COSMOS trial; – Older adults (n = 958; mean age 70.2 ± 5.6 years, 482 women/476 men; 89.1% white)	– Daily multivitamin-multimineral and/or cocoa extract (500 mg cocoa flavanols/day, including 80 mg (−) epicatechin) for 2 years; – Placebo-controlled factorial comparison	Blood, Illumina methylation array	PCHannum, PCHorvath, PCPhenoAge, PCGrimAge and DunedinPACE	– Multivitamin-multimineral arm: PCPhenoAge yearly-change difference −0.214 years/year (95% CI −0.410 to −0.019; *p* = 0.032), Cohen’s d = −0.038 (95% CI −0.074 to −0.003; PCGrimAge difference −0.113 years/year (95% CI −0.205 to −0.020), d = −0.033 (95% CI −0.061 to −0.006), *p* = 0.017 – Cocoa extract showed no significant effect across the five clocks;
Gensous et al., 2020 [25]	– NU-AGE substudy; – Older adults aged 65–79 years from Italy and Poland (n = 120; 60 recruited in Italy and 60 in Poland; Italy: 27 men/33 women; Poland: 24 men/36 women)	– Mediterranean-like diet for 1 year; – Comparator as baseline-to-follow-up comparison within intervention cohort	Whole blood; Illumina 450K methylation array	Horvath DNAm age, AgeAccel, intrinsic and extrinsic epigenetic age acceleration (IEAA, EEAA)	– Polish participants showed reduced AgeAccel after 1 year (*p* = 0.0312). Polish women showed significant decreases in AgeAccel (*p* = 0.0013; adjusted *p* = 0.008) and IEAA (*p* = 0.007; adjusted *p* = 0.04). mean AgeAccelDiff = −1.47 years and mean IEAADiff = −1.36 years. SD of change NR – EEAA was unchanged
Yaskolka Meir et al., 2021 [26]	– Substudy of a randomized lifestyle/weight-loss trial; – Adults with abdominal obesity or dyslipidemia (n = 120; 110 men/10 women; mean chronological age 48.6 ± 9.3 years); recruited from a research-center workplace in Dimona, Israel	– Low-fat diet vs. Mediterranean/low carbohydrate for 18 months; – Diet arm comparison	Blood; Illumina MethylationEPIC BeadChip/850K CpGs; Illumina iScan	240-CpG methylation age with Horvath sensitivity analysis	– No significant diet-arm difference in ΔmAge: 0.9 ± 1.9 years in MED/LC vs. 1.3 ± 1.9 years in LF, *p* = 0.2. – Significant attenuation was observed in participants with >5% weight loss: Δ = 0.6 vs. 1.1 years, *p* = 0.04; and in participants with healthy liver fat: Δ = 0.6 vs. 1.8 years, *p* = 0.003 – Subgroup SD NR
Perlmutter et al., 2024 [27]	– Single-arm open-label pilot intervention; – Adults aged 18–85 years (n = 50 enrolled; 40% men/60% women; n = 47 paired samples; n = 40 per-protocol participants); geographical background was not stated	– Tartary buckwheat-derived polyphenol for 90 days; – No placebo	Blood; Illumina EPICv1 methylation array	OMICmAge; PCPhenoAge; PCGrimAge; DunedinPACE and immune-age markers	– No consistent whole-cohort benefit – Selected subgroup- and clock-specific changes were reported, including changes in PCPhenoAge; OMICmAge and PCGrimAge. – For the entire cohort PCPhenoAge EAA: −0.476 ± 5.580 → −0.262 ± 4.854, *p* = 0.690
Sae-Lee et al., 2018 [28]	– Secondary analysis of two nutritional intervention datasets; – Folic acid + vitamin B12 dataset (n = 44; 19 men/25 women); – Flavanol dataset (n = 13; 13 men/0 women); geographical background was not stated	– Folic acid 400 µg/day + vitamin B12 500 µg/day for 2 years; – Monomeric/oligomeric flavanols, 200 mg for 8 weeks; – Placebo or comparator arms from source datasets	Blood; Illumina Infinium 450K methylation microarray	Horvath DNAm age and epigenetic age acceleration residuals.	– Folic acid + vitamin B12 dataset: reduction in age acceleration only in women with the MTHFR 677CC genotype; effect/SD NR – Flavanol dataset: no significant clock effects
Campisi et al., 2025 [29]	– Randomized, double-blind, placebo-controlled trial; – Adults aged 45–65 years in a monocentric University of Padua/Italy clinical trial setting (n = 81; intervention group: 20 men/20 women; placebo group: 20 men/21 women)	– *Monarda didyma* L. extract, 100 mg/day for 12 weeks; – Maltodextrin placebo	Whole blood; targeted bisulfite pyrosequencing	Targeted five-marker DNAmAge estimator based on ELOVL2, C1orf132, KLF14, TRIM59 and FHL2	– DNAmAge remained stable in the intervention group (*p* = 0.4522), but increased in placebo group (*p* < 0.0001), post-treatment DNAmAge was significantly higher in placebo than intervention (*p* = 0.0162), ELOVL2 increased in placebo (*p* < 0.0001), and post-treatment ELOVL2 and FHL2 methylation were higher in placebo (*p* = 0.0452),
Pastor et al., 2024 [30]	– Prospective before-after study; – Wine consumers aged 40–80 years with cardiovascular risk from Buenos Aires, Argentina (n = 30; 22 men/8 women; median age 66.63 ± 10.14 years)	– Resveratrol-enriched Malbec wine 250 mL/day for men and 125 mL/day for women; enriched to 150 mg/L resveratrol for 105 ± 12.4 days; – No control group	Blood DNA methylation; platform not specified	DNAm PhenoAge and epigenetic age acceleration	– Reduced DNAm PhenoAge; paired-test *p* = 0.0111, r = 0.825. The authors reported deceleration of epigenetic age acceleration by 4.72 years, representing 114% benefit; SD for clock change NR
Minami et al., 2025 [31]	– Secondary longitudinal analysis of public GEO dataset GSE190540; – 20 cognitively normal controls and 25 patients with MCI from Massachusetts General Hospital, USA; 9 women/11 men; MCI: 13 women/12 men; mean age ~70 years	– Vitamin D and/or marine omega-3 fatty acid supplementation for 2 years; – Public longitudinal pre/post dataset with MCI and control comparisons	Peripheral-blood DNA; bisulfite converted DNA; Illumina Infinium MethylationEPIC/850K	HorvathAge, HannumAge, SkinBloodAge, PhenoAge, DunedinPACE, DNAmTL; GrimAge components and GrimAge2	– AgeAccelPheno: 0.975 ± 5.153 → −0.636 ± 4.857 years (*p* = 0.0115; q = 0.0458); AgeAccelGrim: 0.429 ± 2.937 → −0.517 ± 3.159 years (*p* = 0.00807; q = 0.0458); AgeAccelGrim2: 0.396 ± 3.339 → −0.495 ± 3.455 years (*p* = 0.0255; q = 0.0680);
McGee et al., 2024 [32]	– Open-label uncontrolled intervention; – Older adults aged ≥ 60 years (n = 83 participants had baseline samples and 80 had 12-week samples; mean age 71.85 ± 6.23 years; 31 men/49 women; 75 white/5 non-white)	– Multi-component supplements containing vitamin B3, vitamin C, vitamin D, omega-3 fish oil, resveratrol, olive fruit phenols and astaxanthin for 12 weeks – No placebo or parallel control group	Whole blood and saliva; Illumina EPIC/850K methylation array	Horvath; Hannum; DNAm PhenoAge; GrimAge; mean epigenetic age and saliva InflammAge	– No significant whole-cohort change in blood-based clocks or saliva InflammAge – Among participants with baseline InflammAge acceleration ≥ 2 years (*n* = 29), InflammAge decreased by 4.055% (3.31 years; *p* = 0.015) and InflammAge acceleration decreased 46.77% (3.47 years; *p* = 0.0058); SD NR
Carreras-Gallo et al., 2025 [33]	– Single-arm clinical trial; – Adults aged 54–84 years (*n* = 51; 25 women/26 men at baseline); geographical background was not stated	– SRW Cel System multi-ingredient nutraceutical range for 12 months; – No control group	Whole blood; bisulfit-converted DNA, Infinium HumanMethylationEPIC BeadChip	Multiple blood and sputum methylation clocks and biomarkers proxies: PC-Horvath, Horvath skin/blood, Hannum, PhenoAge, GrimAge, OMICmAge, DunedinPACE, DNAmTL, CausAge, DamAge and AdaptAge	– Mixed clock responses: PC-Horvath pan-tissue age and DNAmGrip decreased, DamAge decreased transiently between 3 and 6 months, whereas PC-Horvath skin/blood and DunedinPACE increased by 12 months. – SDs NR
Robinson et al., 2026 [34]	– Randomized crossover-controlled trial; – Adults aged 50–65 years with BMI > 30 kg/m^2^ recruited in the Auburn/Opelika, Alabama, USA (*n* = 21 began, 19 completed; 65% female; baseline table *n* = 20 with 14 women/6 men; *n* = 15 had methylation data)	– Green-based supplements contain dehydrated vegetables, fruits, botanicals, algae, grasses, micronutrients and plant extracts for 30 days – No placebo	Peripheral blood mononuclear cells; Illumina MethylationEPIC array	Horvath, PCGrimAge, AdaptAge and DamAge	– Horvath epigenetic age significantly increased by 1.43 ± 3.61 years during supplementation vs. a decrease of 2.07 ± 4.22 years during control (treatment main effect, *p* = 0.015). – PCGrimAge, AdaptAge and DamAge showed no significant effect
Fitzgerald et al., 2021 [35]/ Villanueva et al., 2025 [36]	– Pilot randomized diet/lifestyle trial and secondary dietary analysis; – Healthy adult men aged 50–72 years from Portland, Oregon, USA (*n* = 43 randomized; 38 analyzed; 81% white)	– Multimodal diet/lifestyle: plant-rich foods, methyl-adaptogen foods, exercise, sleep guidance and meditation for 8 weeks; – Usual-care control	Saliva; Illumina Methylation EPIC array	Horvath 2013 pan-tissue clock	– 3.23 year between-group reduction in Horvath 2013 DNAmAge vs. usual care (*p* = 0.018); – Secondary analysis: methyl-adaptogen intake was associated with greater epigenetic age reduction (B = −1.21 years, 95% CI −2.80 to −0.08; *p* = 0.016) after adjustment for baseline EAA and weight change

AgeAccel: epigenetic age acceleration; BMI: body mass index; CENTRAL MRI: CENTRAL magnetic resonance imaging substudy; DNAm: DNA methylation; DNAmTL: DNA methylation-based telomere length; EAA: epigenetic age acceleration; EEAA: extrinsic epigenetic age acceleration; IEAA: intrinsic epigenetic age acceleration; MCI: mild cognitive impairment; MTHFR: methylenetetrahydrofolate reductase. Effect estimates are presented as reported in the original studies. Standard deviation (SD), standard error (SE), 95% confidence interval (CI) or *p* values are shown when available. NR, not reported.

## Data Availability

Data are available upon reasonable request.

## Supplementary Materials

The following supporting information can be downloaded at https://www.mdpi.com/article/10.3390/antiox15091075/s1, Table S1. Risk of bias and qualitative certainty of evidence across included studies; Table S2. PRISMA 2020 checklist for the present systematic review.
