# Supplementary material for: Natural-Product-Derived Antioxidants and DNA Methylation-Based Epigenetic Aging: A Systematic Review of Human Intervention Studies
Source: Antioxidants (Basel). 2026 Aug 28;15(9):1075. doi: 10.3390/antiox15091075 (PMC13603410; doi:10.3390/antiox15091075)
Supplement: Supplementary file 1 [file antioxidants-15-01075-s001.zip › antioxidants-4495939-supplementary.pdf]

Each study was evaluated for major design limitations, clock-related limitations, attribution concerns, overall risk of bias and certainty of evidence for methylation-aging inference. Risk-of-bias judgments were categorized as low, some concerns or high. Certainty of evidence was summarized qualitatively as strong, moderate, low, very low or intermediate categories when appropriate, according to study design, comparator structure, sample size, directness of the intervention-clock relationship, clock validity, consistency of findings and biological interpretability.

Table S1. Risk of bias and qualitative certainty of evidence across included studies

Study	Main design limitations	Clock-related limitations	Attribution concerns	Overall risk of bias	Certainty of evidence for methylation-aging inference
Sae-Lee et al., 2018	Limited methylation sample size, particularly in the flavanol dataset.	Horvath clock only; limited ability to compare responses across clock families.	Effects were genotype- and sex-specific; flavanol exposure did not produce a whole-cohort clock effect.	High	Low
Gensous et al., 2020	Methylation analysis was mainly intervention pre/post and lacked an independent comparator-supported methylation analysis.	Primarily Horvath-derived DNAm age, AgeAccel, IEAA and EEAA.	Country- and sex-specific signal; whole-diet matrix prevents attribution to polyphenols alone.	Some concerns	Moderate-to-low
Yaskolka Meir et al., 2021	Methylation-aging analysis was secondary.	Validated 240-CpG methylation-age formula with Horvath sensitivity analysis; not a broad modern clock panel.	Methylation-aging attenuation was linked more strongly to weight loss and liver-fat status than to assigned diet arm.	Some concerns	Low-to-moderate
Yaskolka Meir et al., 2023	Methylation findings were partly adherence- and biomarker-associated rather than broad between-arm effects.	Broad clock panel including first-generation, second-generation and pace-of-aging measures.	Polyphenol exposure was embedded in whole-diet change; effects were not uniform across clocks.	Some concerns	Moderate-to-strong
Pastor et al., 2024	Small sample; no control group.	DNAm PhenoAge only.	Resveratrol-enriched wine includes an alcoholic matrix and uncontrolled dietary/lifestyle context.	High	Very low-to-low
McGee et al., 2024	Open-label uncontrolled intervention.	Multiple blood clocks plus saliva InflammAge; whole-cohort effects were mostly null.	Multi-component supplement; signal restricted to participants with elevated baseline epigenetic age or inflammatory burden.	High	Low
Fitzgerald et al., 2021/ Villanueva et al., 2025	Small pilot randomized trial and secondary dietary analysis; male-only and demographically homogeneous sample; dietary intake was self-reported	Horvath 2013 pan-tissue clock only; saliva sample.	Multimodal diet, exercise, sleep and meditation intervention; methyl-adaptogen analysis was exploratory and self-reported.	High	Low
Perlmutter et al., 2024	No placebo control	Multiple clocks were assessed, but findings were heterogeneous and subgroup driven.	Commercial polyphenol supplement; limited causal attribution to the intervention.	High	Very low-to-low

Campisi et al., 2025	Modest sample size and short duration.	Targeted five-marker DNAmAge estimator rather than a genome-wide clock panel.	Botanical extract was more specific than whole-diet interventions, but the endpoint is not directly comparable with genome-wide clocks.	Some concerns	Moderate
Bischoff-Ferrari et al., 2025	Methylation aging was not the original primary endpoint.	Strong clock panels include PhenoAge, GrimAge, GrimAge2 and DunedinPACE.	Omega-3 effect was assessed within a vitamin D and exercise factorial context; the randomized factorial design strengthened interpretation, but methylation aging was not the original primary endpoint.	Some concerns	Strong
Carreras-Gallo et al., 2025	No placebo control; lifestyle advice included	Very broad clock/biomarker panel with discordant responses.	Multi-ingredient commercial formulation plus walking and mindfulness recommendations; active exposure cannot be isolated.	High	Very low-to-low
Minami et al., 2025	Small disease-specific sample.	Multiple clocks and immune-cell estimates were assessed, but the most interpretable intervention signals were mainly for PhenoAge, GrimAge and natural killer cell estimates; small sample size and cell-composition sensitivity limit interpretation.	Vitamin D and omega-3 effects were not clearly separable; mild cognitive impairment population limits generalizability.	High	Very low-to-low
Li et al., 2026	Methylation aging was not the main parent-trial endpoint.	Strong clock panels include PCHannum, PCHorvath, PCPhenoAge, PCGrimAge and DunedinPACE.	Cocoa and multivitamin arms were separable; cocoa null result strengthens interpretability for isolated cocoa flavanol exposure.	Some concerns	Moderate
Robinson et al., 2026	Small randomized crossover-controlled trial; open-label; no placebo; short intervention duration.	PBMC methylation with Horvath, PCGrimAge, AdaptAge and DamAge; limited power due to small methylation sample.	Greens supplement contained many bioactives; adherence was not objectively biomarker confirmed.	High	Low

AgeAccel, epigenetic age acceleration; DNAm, DNA methylation; EEAA, extrinsic epigenetic age acceleration; IEAA, intrinsic epigenetic age acceleration; MCI, mild cognitive impairment; PBMC, peripheral blood mononuclear cell; PC, principal component.

Table S2. PRISMA 2020 checklist for the present systematic review.

Section and topic	Item #	PRISMA 2020 checklist item	Location in manuscript
**TITLE**	1	Identify the report as a systematic review.	Title page.
**ABSTRACT**	2	Provide a structured summary including background, objectives, eligibility criteria, information sources, included studies, synthesis methods, results, limitations and conclusions.	Abstract.
**INTRODUCTION**	3	Describe the rationale for the review in the context of existing knowledge.	Section 1, Introduction.
	4	Provide an explicit statement of the objective(s) or question(s) addressed.	Section 1, final paragraph.
**METHODS**	5	Specify the inclusion and exclusion criteria for the review and how studies were grouped for synthesis.	Section 2.2, Eligibility criteria; Section 2.5, Data synthesis.
	6	Specify all databases, registers, websites, organizations, reference lists and other sources searched or consulted, and specify the date when each source was last searched or consulted.	Section 2.1, Search strategy and study identification.
	7	Present the full search strategy for all databases, registers and websites, including filters and limits used.	Section 2.1; core Boolean search structure provided, with search strings adapted to each database.
	8	Specify the methods used to decide whether a study met the inclusion criteria, including title/abstract screening and full-text eligibility assessment.	Sections 2.1 and 2.3; Figure 1.
	9	Specify the methods used to collect data from reports, including standardized extraction procedures and any processes for confirming data.	Section 2.3, Study selection and data extraction.
	10	List and define all outcomes and other variables for which data were sought, including study characteristics, intervention details, methylation platforms, clock outcomes and secondary biological outcomes.	Sections 2.2 and 2.3; Table 1.
	11	Specify the methods used to assess risk of bias in included studies.	Section 2.4, Risk-of-bias assessment; Table S1.
	12	Specify effect measures used for each outcome.	Not applicable to pooled effect estimation. Outcomes were summarized narratively as clock-related changes, age acceleration measures and direction of effects (Sections 2.2 and 2.5).
	13	Describe the methods used to decide which studies were eligible for each synthesis,	Section 2.5, Data synthesis; Section 3; Table 1; Figure 2.

		prepare data for synthesis, tabulate or visually display results, and synthesize findings.	
	14	Describe any methods used to assess risk of bias due to missing results, including publication bias or selective reporting.	No formal assessment was performed; publication bias and selective outcome reporting are discussed as limitations in Section 5.1.
	15	Describe any methods used to assess certainty or confidence in the body of evidence.	Section 2.4; qualitative certainty ratings reported in Table S1.
**RESULTS**	16	Describe the results of the search and selection process, from records identified to studies included, ideally using a flow diagram.	Section 2.1; Figure 1, PRISMA 2020 flow diagram.
	17	Cite each included study and presenting its characteristics.	Section 3; Table 1.
	18	Present assessments of risk of bias for each included study.	Table S1; summarized in Section 2.4.
	19	For each included study, present results for all outcomes relevant to the review question.	Table 1; Sections 3.1-3.5.
	20	Present results of each synthesis, including consistency, direction of effects and any subgroup or clock-specific patterns.	Sections 3.1-3.5 and Section 4.
	21	Present assessments of risk of bias due to missing results, including publication bias or selective reporting.	No formal assessment was performed; limitations are discussed in Section 5.1.
	22	Present assessments of certainty or confidence in the body of evidence.	Table S1; Sections 3 and 5.1.
**DISCUSSION**	23	Provide a general interpretation of the results in the context of other evidence, discuss limitations of the evidence and review process, and describe implications for practice, policy or future research.	Section 5, Discussion and perspective; Section 5.1, Strengths and limitations; Section 6, Conclusion.
**OTHER INFORMATION**	24	Provide registration information for the review, including register name and registration number, or state that the review was not registered.	Section 2.1: no protocol was registered in PROSPERO.
	25	Describe sources of financial or non-financial support for the review and the role of funders or sponsors.	Funding statement
	26	Declare any competing interests of review authors.	Conflicts of Interest section.
	27	Report on availability of data, code and other materials used in the review.	Data Availability Statement; Supplementary Materials section; Table S1 and Table S2.
